# Coronary magnetic resonance angiography: in vivo comparison of image quality at 1.5 Tesla versus 3.0 Tesla with Parallel Radiofrequency Transmission

**DOI:** 10.1186/1532-429X-14-S1-P255

**Published:** 2012-02-01

**Authors:** Tarique Hussain, Khalid Hussain, Sarah A Peel, Gerald F Greil, Rene M Botnar, Andrea Wiethoff

**Affiliations:** 1 Division of Imaging Sciences, King's College London, London, UK; 2 Philips Healthcare (UK), Guildford, UK; 3 Surgery, Russells Hall Hospital, Dudley, UK

## Summary

Coronary plaque and thrombus characterization at 3.0 Tesla holds great potential for clinical benefit but problems still exist with coronary lumen image quality at 3.0 Tesla, which is a pre-requisite for these techniques. This study shows that despite implementation of parallel transmit technology to improve radiofrequency (B1 field) homogeneity, balanced steady-state-free-precession (b-SSFP) Coronary Magnetic Resonance Angiography (CMRA) at 3.0 Tesla is still inferior to 1.5 Tesla imaging. Parallel transmit will need to be combined with other recent technological advances to achieve superior imaging at 3.0 Tesla.

## Background

3.0 Tesla field strength has been shown to be advantageous for coronary plaque and thrombus characterization. However, good coronary lumen imaging is a pre-requisite for this type of imaging. b-SSFP has been shown to have superior contrast-to-noise ratio (CNR) and signal-to-noise ratio (SNR). However, it is susceptible to field inhomogeneity at high field strengths. Parallel radiofrequency transmission (Tx) can improve B1 field homogeneity and reduce B1 attenuation artifacts. The purpose of this study is to investigate if, in comparison to 1.5 Tesla imaging, b-SSFP CMRA image quality at 3.0 Tesla can be maintained by use of Tx.

## Methods

Ten subjects (7 male) underwent b-SSFP CMRA at 3T and 1.5T (Achieva, Philips Healthcare, Best, Netherlands). 32-element cardiac phased-array receiver coil was used. Targeted CMRA of the left coronary system was performed using free-breathing, cardiac-triggered, T2-prepared, 3d b-SSFP. At 3.0T, a B1 volume shim, utilizing Tx technology, was applied. Parameters included: FOV=270x270mm, resolution=1.25x1.25mm, slice thickness=3mm overcontiguous, acquisition in mid-diastole, TR/TE=5.2/2.6ms, FA=90° (1.5T) & 70° (3.0T). Analysis used “Soap-Bubble” software (Philips Medical Systems). Proximal vessel diameter; SNR; CNR; vessel sharpness; vessel length; number of proximal left anterior descending (LAD) branches imaged; number of left coronary artery segments (AHA classification) imaged and consensus reading for qualitative image quality (IQ) (score 0 to 4, McConnell et al, 1997) were measured. Paired t-tests were used for comparison but non-parametric variables were compared using Wilcoxon Signed Ranks Test.

## Results

As expected, SNR values were significantly higher at 3.0 Tesla (33 vs. 23; p=0.01) and there was a trend towards higher CNR (17 vs. 14; p=0.06). This resulted in better distal visualization with a greater visualized length of LAD at 3.0T (8.4 vs. 7.1cm; p=0.03). It also resulted in non-inferiority when compared with 1.5 Tesla for first-order branch visualization (median 2.5 at 3.0T and 2.0 at 1.5T; p=0.92) and for number of segments seen (median for both = 10; p=0.86).

**Figure 1:**
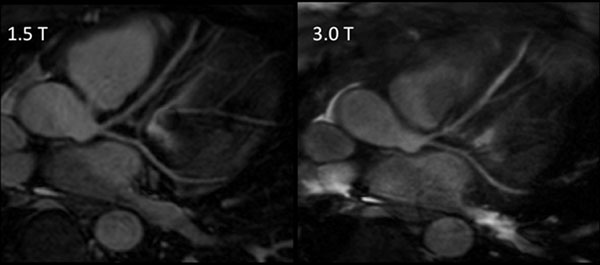
Sample Images showing left coronary system of same volunteer at 1.5 Tesla (T) on left and 3.0 T on right. Images have been reformatted using “Soap-Bubble”

**Figure 2:**
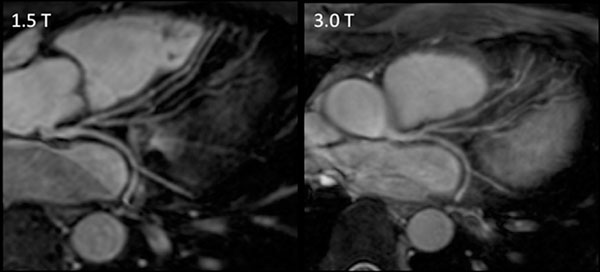
Sample Images showing left coronary system of same volunteer at 1.5 Tesla (T) on left and 3.0 T on right. Images have been reformatted using “Soap-Bubble”

However, image quality was worse quantitatively by vessel sharpness (37% at 3.0T and 43% at 1.5T; p=0.011) and qualitatively by IQ (median 3.0 at 3.0T and 4.0 at 1.5T; p=0.011). The reduction in image quality resulted in an overestimation of vessel size at 3.0 Tesla in comparison to 1.5 Tesla (mean bias = 0.2mm; p=0.049; 95% limits of agreement on Bland-Altman analysis were 0.35 to -0.75mm). (Sample images given in Figure 1 and 2)

## Conclusions

Coronary plaque and thrombus characterization at 3.0 Tesla holds great potential for clinical benefit but problems still exist with coronary lumen image quality at 3.0 Tesla. This study shows that despite implementation of parallel transmit technology, b-SSFP CMRA at 3.0 Tesla is still inferior to 1.5 Tesla imaging. Parallel transmit should be combined with other recent technological advances to achieve superior imaging at 3.0 Tesla.

## Funding

We would like to thank the Well Child Foundation (Cheltenham, UK) for financial support of the Senior Clinical Lecturer position of Gerald F. Greil, MD. The authors acknowledge financial support from the Department of Health via the National Institute for Health Research (NIHR) comprehensive Biomedical Research Centre award to Guy’s & St Thomas’ NHS Foundation Trust in partnership with King’s College London and King’s College Hospital NHS Foundation Trust. The MRI scanner is partly supported by Philips Healthcare. A. Wiethoff is an employee of Philips Healthcare, Best. All the other authors were not consultants or employees for Philips Healthcare and had control of inclusion of any data and information that might present a conflict of interest for A. Wiethoff.

No financial support was provided for data collection and analysis of manuscript preparation.

